# Evaluation of Reference Genes for Gene Expression Analysis Using Quantitative RT-PCR in *Azospirillum brasilense*

**DOI:** 10.1371/journal.pone.0098162

**Published:** 2014-05-19

**Authors:** Mary McMillan, Lily Pereg

**Affiliations:** School of Science and Technology, University of New England, Armidale, New South Wales, Australia; Harvard Medical School, United States of America

## Abstract

*Azospirillum brasilense* is a nitrogen fixing bacterium that has been shown to have various beneficial effects on plant growth and yield. Under normal conditions *A. brasilense* exists in a motile flagellated form, which, under starvation or stress conditions, can undergo differentiation into an encapsulated, cyst-like form. Quantitative RT-PCR can be used to analyse changes in gene expression during this differentiation process. The accuracy of quantification of mRNA levels by qRT-PCR relies on the normalisation of data against stably expressed reference genes. No suitable set of reference genes has yet been described for *A. brasilense*. Here we evaluated the expression of ten candidate reference genes (*16S rRNA, gapB, glyA, gyrA, proC, pykA, recA, recF, rpoD, and tpiA*) in wild-type and mutant *A. brasilense* strains under different culture conditions, including conditions that induce differentiation. Analysis with the software programs BestKeeper, NormFinder and GeNorm indicated that *gyrA*, *glyA* and *recA* are the most stably expressed reference genes in *A. brasilense*. The results also suggested that the use of two reference genes (*gyrA* and *glyA*) is sufficient for effective normalisation of qRT-PCR data.

## Introduction

*Azospirillum brasilense* is a facultative aerobic, nitrogen-fixing bacterial species, which is associated with the rhizosphere of various plants. *A. brasilense* strains excrete plant hormones that have beneficial effects on both plant growth and yield [1], [2] and have been implicated in disease suppression [3, and L Pereg, unpublished]. Under normal conditions *A. brasilense* strains exist in a flagellated, motile form. Under stress or nutrient-limiting conditions *A. brasilense* strains are able to change their metabolic activity and undergo differentiation into encapsulated cyst-like forms [4], [5]. Such differentiation is an essential step for *A. brasilense* survival under adverse conditions and for firm attachment to plant roots [6], [7]. It may influence other processes while in association with plants, such as nitrogen fixation [8]. Its beneficial properties and the various modes of interaction with plant roots make this species a model organism for studying bacterial-plant interactions. Understanding the expression pattern of genes involved in various processes undertaken by *Azospirillum* should provide useful information about the regulatory networks involved in cellular differentiation, hormonal production, nitrogen and carbohydrate metabolism, just to name a few.

Quantitative reverse transcribed polymerase chain reaction (qRT-PCR) has become the preferred method for the study of differential mRNA expression [9]. qRT-PCR is highly sensitive and specific, and is therefore a powerful technique for monitoring changes in the expression of genes during processes such as cellular differentiation [10], [11]. qRT-PCR can be used as both an independent method for expression analysis, and as a method to confirm results of differential gene/protein studies obtained by microarray or proteomics analysis [12], [13]. The accuracy of quantification of relative mRNA levels by qRT-PCR relies on the normalisation of data against internal reference genes (i.e. genes that are stably expressed under the various experimental conditions) [14], [15]. Reference genes are used to eliminate sample to sample variation. It is therefore essential to identify stably expressed reference genes, as variation in reference gene expression can create false positives, or mask real positives [16]. It is generally recommended to use more than one reference gene to obtain accurate normalisation of data [15], [17], [18].

In eukaryotic cells, a number of stably expressed reference genes have been identified that can be used for routine normalisation during quantitative expression analysis, including β*-actin* and *GAPDH* [18], [19]. However, no such standard set of reference genes has been determined for prokaryotic cells, as expression of typical reference genes in prokaryotes has been shown to be highly variable under different experimental conditions [20]. Previous studies have employed *16S rRNA* as a reference gene for normalisation of gene expression data [21], [22]. However, *rRNA* expression has been shown to be highly dependent on the physiological status of the bacterial cell [20], [23]. Thus, for bacteria such as *A. brasilense*, which undergoes differentiation in response to stress, *16S rRNA* may not be a reliable reference gene. There is therefore a need to identify reference genes that are expressed stably in *A. brasilense* in both its motile and cyst-like forms. To date there has been no systematic analysis carried out to identify suitable reference genes for normalization of qRT-PCR data in *A. brasilense*.

Wild-type strains Sp7 and Sp245 show some metabolic differences as well as different patterns of plant interactions, with both attaching to the root surface but only Sp245 internally colonising the root system [24]. Strains Sp7 and Sp245 can undergo differentiation into cyst-like forms and flocculate in stress-inducing medium [6]. Moreover, wild-type and non-flocculating mutant strains of *A. brasilense* affected in differentiation, show major differences in both morphology and physiology when grown under various conditions [6], [7], [8]. They are expected to have overall different gene expression patterns under such conditions, making them suitable for use in the selection of reference genes for qRT-PCR experiments. We have cultured wild-type and mutant strains in different growth media, including media that induces flocculation in the wild types, to test the expression stability of ten reference gene candidates and determine which are the most suitable for use as reference genes for qRT-PCR experiments. The reference gene candidates tested were *16S rRNA*, *gapB (GAPDH)*, *glyA*, *gyrA*, *proC*, *pykA*, *recA*, *recF*, *rpoD* and *tpiA*. The programs BestKeeper [25], NormFinder [26] and GeNorm [17] were used to analyse the stability of the candidate genes. We report here on reference genes suitable for effective normalisation of qRT-PCR data for various *A. brasilense* strains.

## Materials and Methods

### Selection of candidate genes and primer design

Ten candidate reference genes (*rpoD*, *gapB*, *16S rRNA*, *glyA*, *recA*, *proC*, *gyrA*, *recF*, *pykA* and *tpiA*) were selected from genes previously used in quantitative RT-PCR assays of other bacterial species (Table 1). Primers were designed using Primer3 software (http://frodo.wi.mit.edu/) based on the available DNA sequences of *A. brasilense* Sp245 (GenBank Assembly ID GCA_000237365.1). Primer efficiencies were determined by construction of a standard curve using 5-fold serial dilutions of pooled cDNA template. Primer specificity was determined by melt curve analysis and gel electrophoresis.

**Table 1:** qRT-PCR primer sets used in this study.

Gene	Forward Primer (5′-3′)	Reverse Primer (5′-3′)	Amplicon size (bp)	Efficiency (%)	Correlation coefficient (R^2^)
*16S rRNA*	ACACATGCAAGTCGAACGAG	CGTCCGTTTCCAGACGTTAT	100	86	0.996
*gapB*	CTTCGTGCTGAACAAGCTGA	AGGTCCTTGTGGTTGGTGTC	102	109.1	0.997
*glyA*	GGAGATCGCCAAGAAGATCA	GCTCTTGGCGTAGGTCTTGA	133	104	0.998
*gyrA*	TCACCGACGAAGAGTTGATG	CTCTTCGATCTCGGTCTTGG	143	98.4	0.999
*proC*	CAAAACCATCGCCTCCTTC	AATCGCACAGCGACTTCTG	149	106.6	0.988
*pykA*	GACCTTTACCGCACGATGAT	TGGTGGTGTAGGTGACGATG	130	101	0.996
*recA*	GTCGAACTGCCTGGTGATCT	GACGGAGGCGTAGAACTTCA	112	95.2	0.997
*recF*	GCTGTTCGACGAGATCCTG	CTCGATGTGGAAGCGTTTG	106	109.1	0.996
*rpoD*	CGTCACCTATGACGAGCTGA	CTCTTCCGATTCGACGATGT	118	102.2	0.998
*tpiA*	GGTCCTCTACGGCGGTTC	AGAAATCGTCCGCCTTCAG	110	96.8	0.999

### Bacterial strains and culture conditions

Three strains of *A. brasilense* were used in this study: wild-type *A. brasilense* Sp245, wild-type *A. brasilense* Sp7, and a non-flocculating *flcA* deletion mutant Sp7-flcAΔ (X Hou and L Pereg, unpublished), which is similar in phenotype to the *flcA* Tn5-induced mutant Sp72001 [6]. *A. brasilense* strains were grown aerobically at 30°C, 180 rpm, in nutrient broth medium (NB; Difco) or nitrogen-free medium (NFB) [27]. Flocculation tests were performed as previously described [27]. Briefly, cultures were first grown in NB medium to an A_600_ of 0.8–0.9 and the cells harvested by centrifugation at 10,000× g for 1 min. The pellet was washed in minimal medium [27] and then used to inoculate 10 mL of flocculation medium (minimal medium supplemented with 8 mM fructose and 0.5 mM KNO_3_) in a 50 ml flask, to an A_600_ of 0.3–0.4. The flasks were incubated with shaking at 200 rpm, 28°C, and checked periodically for flocculation. Sp7 and Sp245 underwent flocculation within 3–4 hours, while the Sp7-flcAΔ mutant fails to undergo flocculation as shown previously for *flcA* Tn5 induced mutants [6].

### RNA extraction, cDNA synthesis and quantitative PCR

Total RNA was extracted from cell samples using a TRIzol Max Bacterial Isolation kit (Invitrogen, USA) and quantified using a NanoDrop ND-1000 spectrophotometer (Thermo Fisher, USA). cDNA was synthesised in random hexamer primed reactions using a SuperScript III first strand synthesis kit (Invitrogen, USA). qRT-PCR reactions were carried out in a Rotor-Gene Q thermal cycler (Qiagen, USA). Each reaction contained 1× IQ SYBR Green Supermix (Bio-Rad, USA), 0.5 µM each forward and reverse primer, and cDNA transcribed from 10 ng RNA. Samples from three independent experiments (biological replicates) were analysed in triplicate (technical replicates), with negative controls included in each assay.

### Data analysis

Expression data for the candidate reference genes was obtained in the form of threshold cycle (Ct) values. The amplification efficiencies and correlation coefficients were calculated using Rotor-Gene Q software (Qiagen, USA). The stability and suitability of reference genes was evaluated using three independent software packages: BestKeeper [25], NormFinder [26] (GenEx version: MultiD, USA) and GeNorm [17] (GenEx version: MultiD, USA). All analyses were carried out using standard setup configurations.

## Results

### Standard curve, PCR efficiency and product specificity

**Figure 1:**
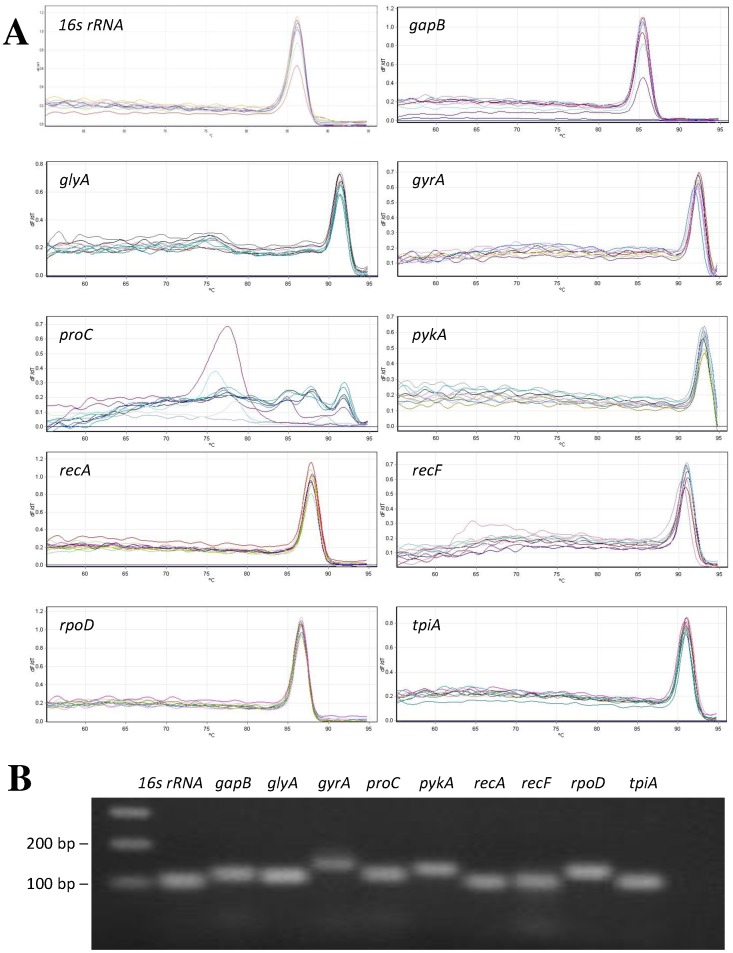
Specificity of qRT-PCR amplification. A: Melt curves for candidate reference genes. B: Agarose gel (2%) showing amplification of a single product of expected size for each candidate reference gene.

The PCR reaction efficiency of each candidate reference gene was determined using a 5-fold serial dilution of pooled cDNA. The calculated efficiencies for the candidate genes, shown in Table 1, were between 95.2 and 109.1%, with the exception of *16S RNA* which showed an amplification efficiency of 86%. Amplification efficiencies greater than 100% may result from inhibition by reverse transcriptase which may result in an overestimate of the “real efficiency” [28]. The efficiency curves for the candidate reference genes were found to have a linear correlation coefficients (R^2^) ranging from 0.988 to 0.999. Melt peak analysis demonstrated a single homogenous peak for all primer sets, with the exception of *proC*, indicating specific amplification of a single product, with no primer-dimer being observed (Figure 1A). The *proC* primer set showed multiple melt peaks, indicating the amplification of multiple non-specific products. Gel electrophoresis analysis of the amplified products for all primer sets revealed single bands of the expected size (Figure 1B). Based on these results the *16S RNA* and *proC* primer pairs were excluded from further analysis.

### Expression of reference gene candidates in culture

**Figure 2:**
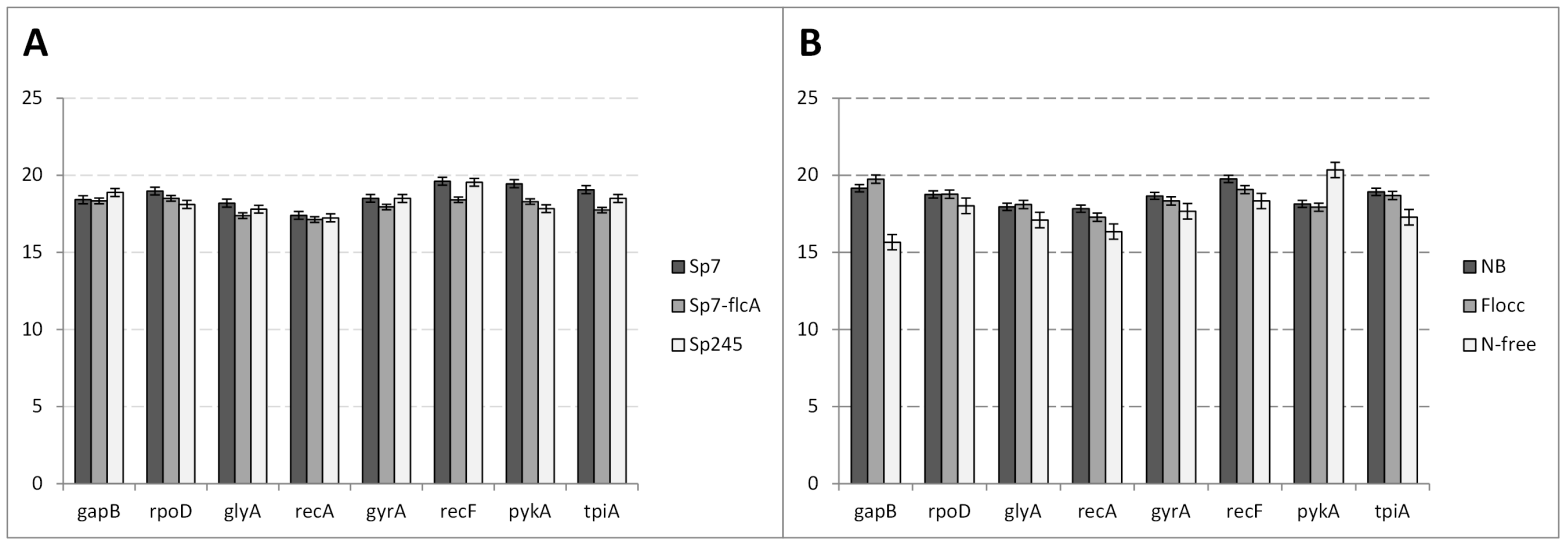
Expression levels of reference gene candidates. A: Expression of reference genes across *A. brasilense* strains. B: Expression of reference genes across different culture conditions. Gene expression levels are represented by average Ct values. NB: nutrient broth; Flocc: Flocculation medium; N-free: Nitrogen free medium. Each bar represents the mean Ct values from three independent experiments. Error bars indicate standard error.

The eight remaining candidate genes showed a relatively narrow range of Ct values. The lowest Ct value was 15.49 (*gapB* expression in Sp7 under N-free conditions), and the highest was 21.1 (*pykA* expression Sp7 under N-free conditions). The majority of the remaining Ct values were between 16 and 20, with the average Ct value of all candidates across all strains and culture conditions being approximately 18.32. The *recA* gene showed the highest average expression across all strains and culture conditions, with an average Ct of approximately 17.25, while *recF* was the least abundant transcript across all strains and culture conditions, with an average Ct of approximately19.14. The transcriptional levels of all candidate genes, as indicated by average Ct, are shown in Figure 2. Figure 2A shows expression of candidate genes across the different strains used. *recA* showed the least variation in Ct values between different strains, with a variation of 0.27, while *pykA* showed the greatest variation in Ct values, with a variation of 1.61. Figure 2B shows expression of candidate genes across different culture conditions. Overall, expression of candidate genes showed greater variation across culture conditions than across strains. *rpoD* showed the least variation in Ct values across culture conditions, with a variation of 0.76, while *gapB* showed the greatest variation in Ct values, with a variation of 4.08.

### Analysis of reference gene expression using BestKeeper, NormFinder and GeNorm

The Excel-based program BestKeeper was used to determine the reference genes with the greatest expression stability. The descriptive statistics of the eight candidate genes are given in Table 2. Based on the BestKeeper analysis the *gyrA* gene was ranked as the most stably expressed gene in the three *A. brasilense* strains across all growth conditions, with a standard deviation (SD) of 0.47. The *glyA* gene was ranked as the second most stably expressed gene, with an SD of 0.53. *gapB* and *pykA* were found to be the least stably expressed genes, with an SD of 1.46 and 0.91 respectively.

**Table 2:** Descriptive statistics of reference gene expression by BestKeeper.

	*gapB*	*rpoD*	*glyA*	*recA*	*gyrA*	*recF*	*pykA*	*tpiA*
geo Mean [CP]	18.42	18.56	17.78	17.24	18.28	19.12	18.57	18.40
ar Mean [CP]	18.50	18.58	17.79	17.25	18.29	19.14	18.61	18.43
min [CP]	15.49	17.55	16.85	15.75	17.06	17.22	16.96	16.41
max [CP]	19.98	20.40	19.45	18.75	19.25	20.15	21.10	19.66
std dev [± CP]	1.46	0.66	0.53	0.65	0.47	0.71	0.91	0.74
CV [% CP]	7.88	3.54	3.00	3.77	2.58	3.71	4.88	4.01
min [x-fold]	−7.61	−2.02	−1.90	−2.80	−2.33	−3.74	−3.06	−3.98
max [x-fold]	2.95	3.57	3.19	2.85	1.96	2.04	5.76	2.39
std dev [± x-fold]	2.75	1.58	1.45	1.57	1.39	1.64	1.88	1.67

Based on inspection of standard deviations (std dev; SD) genes can be ranked from the most stably expressed (lowest SD: *gyrA*), to the least stably expressed (highest SD: *gapB*). Genes with std dev [±CP]>1 are considered to be inconsistent. CP = crossing point or threshold cycle (Ct).

**Figure 3:**
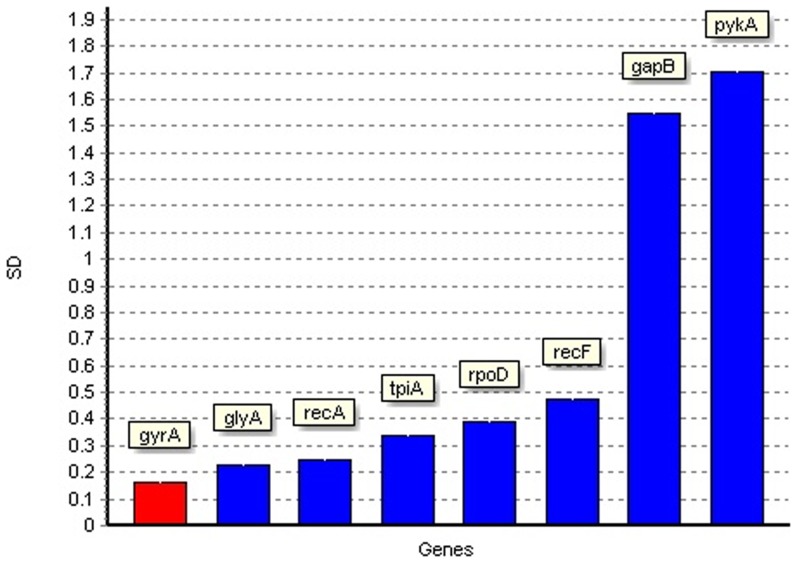
Analysis of reference gene stability by NormFinder. Low standard deviation (SD) values indicate stable gene expression. Genes are ordered left to right in order of decreasing stability.

**Figure 4:**
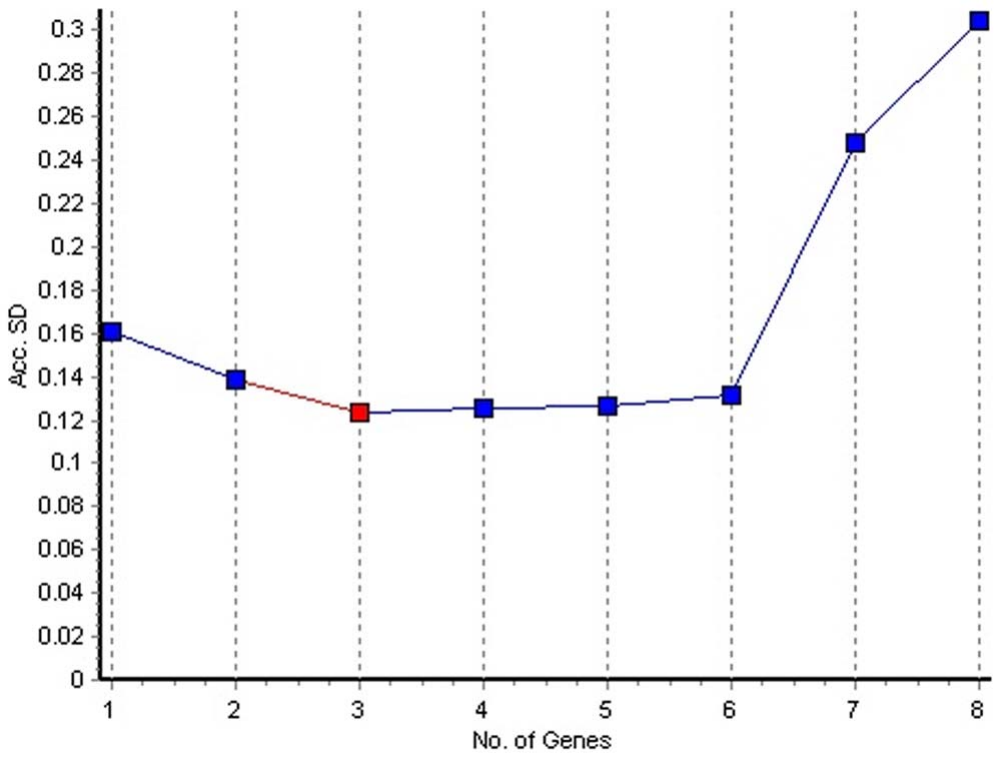
NormFinder estimation of the optimum number of reference genes. Lowest accumulated standard deviation (Acc. SD) values indicate the optimal number of reference genes. The recommended upper limit for Acc. SD is set at 0.15.

The NormFinder program was also used to rank the candidate reference genes according to expression stability across all culture conditions. NormFinder analysis (Figure 3) showed that *glyA* and *gyrA* had the smallest variability, with SD of 0.22 and 0.16 respectively, indicating that they were therefore the most stable genes. The *recA* gene also showed low variability, with an SD of 0.25. The *pykA* and *gapB* genes were found to be the least stable, having the highest variability with stability values of 1.70 and 1.54 respectively. The NormFinder algorithm also calculates the best combination of two reference genes to be used for data normalization. The combination with the lowest variability was found to be *glyA* and *gyrA*. The NormFinder algorithm also calculates the optimal number of reference genes based on Accumulated standard deviation (Acc SD) (Figure 4). The optimal number of reference genes was shown to be 3 (*gyrA*, *glyA* and *recA*), with an Acc SD of 0.13. The combination of 2 genes (*gyrA* and *glyA*) also fell under the suggested value of 0.15.

**Figure 5:**
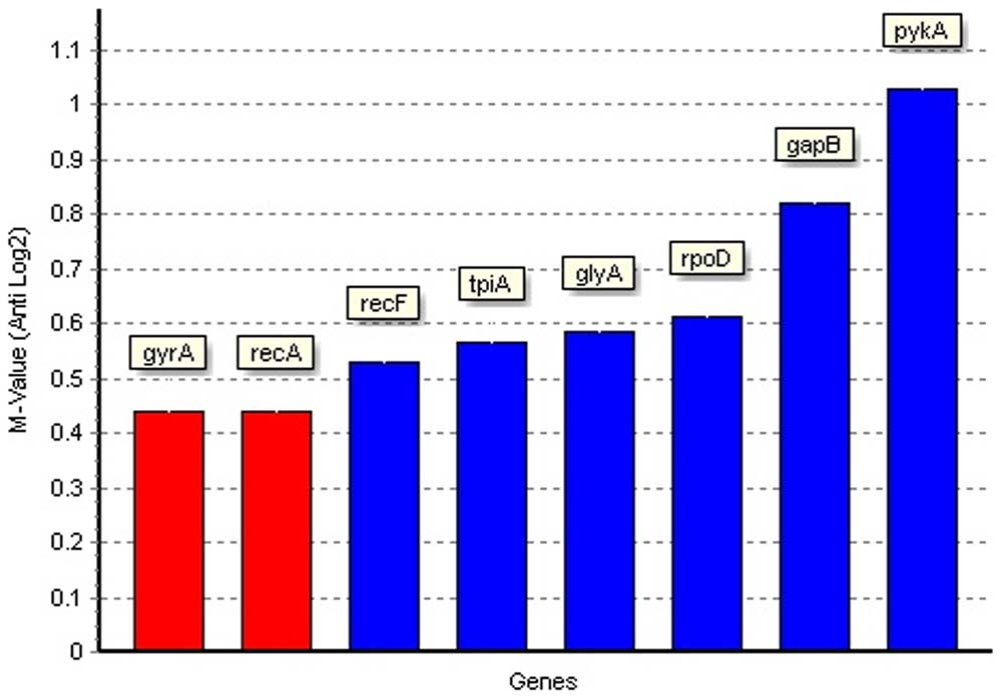
Analysis of reference gene stability by GeNorm. Low stability values (M-values) indicate stable gene expression. Genes are ordered left to right in order of decreasing stability.

Finally, the GeNorm program was also used to rank the candidate reference genes according to their expression stability value, M (Figure 5). The most stable reference genes across all culture conditions, based on lowest M value, were found to be *gyrA* and *recA*, both with an M value of 0.44. The next most stable gene was *recF*, with an M value of 0.53. The *pykA* and *gapB* genes were again found to be the least stable, with M values of 1.03 and 0.82 respectively.

The results of the BestKeeper, NormFinder and GeNorm analyses are summarised in Table 3.

**Table 3:** Summary of reference gene ranking by BestKeeper, NormFinder and GeNorm analysis.

Gene	BestKeeper (SD)	NormFinder (SD)	GeNorm (M value)
*gapB*	1.46+	1.54	0.82+
*glyA*	0.53	0.22	0.58
*gyrA*	0.47*	0.16*	0.44*
*pykA*	0.91	1.70+	1.03
*recA*	0.65	0.24	0.44*
*recF*	0.71	0.47	0.53
*rpoD*	0.66	0.39	0.61
*tpiA*	0.74	0.33	0.56

*Indicates the most stably expressed gene/s and + the least stably expressed gene as determined by each program.

## Discussion

Quantitative RT-PCR has become an important technology for studying differential gene expression. One essential aspect of qRT-PCR studies is the use of highly reliable combinations of reference genes for the normalization of data. No standard set of reference genes has been identified for prokaryotes, as expression of typical reference genes has been shown to vary greatly under different experimental conditions [20]. Here, we describe a set of reference genes that can be used for the normalization of gene expression data in qRT-PCR experiments in different strains of the bacterium *Azospirillum brasilense* in both vegetative motile and cyst-like forms.

Direct analysis of the distribution of Ct values from qRT-PCR experiments cannot be used to rank candidate reference genes according to stability as it fails to take into account PCR efficiencies. Several statistical algorithms have been developed to select stably expressed reference genes. In this study we have used three common software programs (BestKeeper, NormFinder and GeNorm) to determine the most stable reference genes in *A. brasilense* under different culture conditions, including conditions that induce cellular differentiation. The BestKeeper algorithm uses Ct values directly to select the most stably expressed reference gene based on variations in the geometric means of Ct values [25]. The NormFinder and GeNorm algorithms use relative quantities derived from Ct values when calculating stability. NormFinder uses a model-based approach to evaluate the stability of individual reference genes while taking into account variation across subgroups, and avoids the artificial selection of co-regulated genes [26]. The GeNorm algorithm selects an optimal number of reference genes out of a larger group by selecting those that show the most similar expression across groups [17]. At present there is no consensus as to which of these algorithms should be used to select the ideal reference gene. Therefore a comparison of reference genes selected using different algorithms allows a better identification of the most reliable controls and reduces the risk of artificial selection of co-regulated transcripts [29]. The three programs showed a high level of consistency in ranking the most and least stable reference genes. Both BestKeeper and NormFinder ranked *gyrA* and *glyA* as the two most stable reference genes, while the GeNorm analysis gave slightly different results, ranking *gyrA* and *recA* as the two most stable genes. All three programs ranked *gapB* and *pykA* as the two least stable reference gene candidates, with NormFinder and GeNorm ranking *pykA* the least stable and BestKeeper ranking *gapB* as the least stable. These slight differences in ranking are to be expected, as each program uses a distinct statistical algorithm to rank genes according to stability. In addition to ranking genes based on stability, the NormFinder analysis also allowed for determination of the optimal number of reference genes to be used in qRT-PCR experiments. Ideally, based on the results of this study, a combination of three reference genes (*gyrA*, *glyA* and *recA*) is recommended for the normalisation of data. However, the use of the two most stable reference genes (*gyrA* and *glyA*) is also appropriate given that this combination also falls under the suggested Acc SD value of 0.15.

The reference gene candidates analysed in this study were selected from genes previously used in qRT-PCR assays of other bacterial species and included *rpoD* [30], *glyA* [31], *tpiA* [31], [32], *pykA* [31], [32], *recF* [31], *gapB* [33], *gyrA* [30], [32], [34] and *recA* [33], [34]. The results of this study indicate that, of the genes tested, *gyrA*, *glyA* and *recA* are the most stably expressed reference genes in *A. brasilense*. *gyrA* has been identified as a stable reference gene for studies in *Xanthomonas citri* [30], and *glyA* has been verified as an appropriate reference gene for normalisation of qRT-PCR data in *Actinobacillus pleuropneumoniae* [31]. In contrast, Theis et al. [32] found that *gyrA* was not suitable as a reference gene for qRT-PCR studies in *Staphylococcus aureus*. Zhao et al. [33] identified *gapB* as the most stable reference gene in *Lactobacillus casei*, and found that *recA* was the least stable of the genes tested. Our results indicate that the opposite is true for *A. brasilense*, with *gapB* being identified as one of the least stable of the genes tested, and *recA* being one of the most stably expressed reference genes. Takle et al. [34] also found *recA* to be one of the most stably expressed reference genes for normalisation of gene expression data in *Pectobacterium atrosepticum*. These results provide further evidence that expression of standard reference genes can be highly variable in prokaryotes depending on species and experimental conditions. Therefore no standard set of reference genes exists for gene expression studies in prokaryotes. This also highlights the importance of carrying out a reference gene stability study to select the most stable reference genes for a particular species under a given set of experimental conditions.

The reference genes in this study were identified by analysis of two distinct *A. brasilense* strains, Sp245 and Sp7. *A. brasilense* Sp245 is an endophytic strain, capable of colonising both the root interior and exterior surface, while *A. brasilense* sp7 is a non-endophytic strain, colonising only the root surface [24]. The different environmental niches colonised by these stains suggest that their responses to environmental changes and stressors may also differ. In addition a mutant strain of Sp7 that is unable to differentiate into cyst-like forms, flocculate or colonise root surfaces [6] and L Pereg, unpublished] was also studied. These wild type and mutant strains exhibit different physiology under stress conditions that induce flocculation [6], [7], [8]. Reference gene expression in prokaryotes has been shown to vary significantly with experimental conditions, and may vary with the physiological status of the cell. The *A. brasilense* strains and culture conditions used in this study were selected to allow for identification of reference genes that were stably expressed by bacteria in different physiological states. Therefore the reference genes identified in this study are suitable for normalisation of gene expression data not only from different strains of *A. brasilense*, but also *A. brasilense* strains exhibiting different physiological behaviour, as induced by environmental stress. They therefore represent a robust set of genes that should be applicable for data normalization in a wide range of gene expression studies in *A. brasilense*.

As this study was restricted to a detailed analysis of only 8 candidate genes it does not provide an exhaustive list of suitable reference genes. However, the most stably expressed reference genes identified here (*gyrA*, *glyA* and *recA*) are more than sufficient for the normalization of qRT-PCR data in *A. brasilense*, both during normal growth and under stress conditions that trigger cellular differentiation. The reference genes identified in this study may also provide a starting point for selection of candidate reference genes for gene expression studies in other related species.
